# A 4-Year Longitudinal Study of the Sex-Creativity Relationship in Childhood, Adolescence, and Emerging Adulthood: Findings of Mean and Variability Analyses

**DOI:** 10.3389/fpsyg.2018.02331

**Published:** 2018-11-26

**Authors:** Wu-Jing He

**Affiliations:** ^1^Department of Special Education and Counselling, The Education University of Hong Kong, Tai Po, Hong Kong; ^2^Integrated Centre for Wellbeing, The Education University of Hong Kong, Tai Po, Hong Kong

**Keywords:** sex differences, creativity, developmental perspective, longitudinal design, variability analyses

## Abstract

The relationship between sex and creativity remains an unresolved research question. The present study aimed to approach this question through the lens of *the developmental theory of sex differences in intelligence*, which posits a dynamic pattern of sex differences in intellectual abilities from female superiority in childhood and early adolescence to male superiority starting at 16 years of age. A total of 775 participants from three age groups (i.e., children, adolescents, and emerging adults) completed a 4-year longitudinal study comprising four assessments of creative thinking at 1-year intervals. Creative thinking was assessed with the Test for Creative Thinking-Drawing Production. While the results revealed female superiority in childhood and early adolescence, male superiority was not found in adolescence and emerging adulthood. Rather, greater sex similarities and greater male variability were found based on mean and variability analyses, respectively. This study elucidated the link between sex and creativity by (1) taking a developmental perspective, (2) employing a 4-year longitudinal design in three age groups (i.e., children, adolescents, and emerging adults), and (3) analyzing sex differences based on both mean and variability analyses.

## Introduction

Creativity is commonly conceptualized as the ability to produce valuable ideas or problem solutions that have the characteristics of originality and appropriateness (Sternberg and Lubart, 1999). Its value has been well recognized for its wide range of contributions, e.g., social progress, personal growth, and individuals’ mental health (Mohamed, 2014). A large body of research has been conducted to gain a better understanding of creativity (Hennessey and Amabile, 2010). Over the years, the research question with respect to sex differences in creativity has remained intriguing (Abraham, 2016). Joining this line of research, the present study aimed to approach this question through the lens of *the developmental theory of sex differences in intelligence*, which may illuminate the unresolved issue from a developmental perspective.

### The Developmental Theory of Sex Differences in Intelligence

The developmental theory of sex differences in intelligence has been proposed by Lynn (1994, 1999) to understand the sex differences in intellectual abilities in relation to developmental stages, postulating a dynamic pattern of sex differences in intellectual abilities from small sex differences (favoring females) in childhood and early adolescence to increasing sex differences (favoring males) starting at 16 years of age. Lynn et al. (2000) further specified that: “…sex differences in intelligence are small (in favor of females) over the age range 8–15 years, but they begin to increase progressively in favor of males from the age of 16 years onwards until among adults they become appreciable…” (p. 556). The developmental theory of sex differences in intelligence has received much empirical support from the literature on sex and intelligence (Colom and Lynn, 2004; Lynn and Kanazawa, 2011). Generally, these research findings have revealed a sex-linked pattern of intellectual abilities from a female superiority in childhood and early adolescence to a male superiority emerging in adolescence. The most convincing evidence may come from a large-scale longitudinal study conducted by Lynn and Kanazawa (2011), in which more than 14,000 respondents completed three waves of assessment on their intelligence at ages 7, 11, and 16. Consistent with this theory, the longitudinal data illustrated that girls outperformed boys on the IQ test at 7 and 11 years of age. However, when these participants reached the age of 16, they exhibited a reverse pattern, in which boys outperformed girls on the IQ test.

Other cross-sectional studies have also provided empirical support for this theory. For example, Arden and Plomin (2006) conducted a study with a sample of children aged 2, 3, 4, 7, 9, and 10 years and found a pattern of female superiority in the younger age groups, in which girls were significantly overrepresented in the upper tail while boys were overrepresented in the lower tail of the intelligence score distribution. Moreover, Colom and Lynn (2004) conducted a large-scale standardization study of the Differential Aptitude Test (DAT) that involved a sample of adolescents aged 12–18 years and found that girls outperformed boys in the younger age groups. However, among the older age groups, they found that girls’ performance declined relative to that of boys; additionally, a pattern of male superiority was found in the age group of 18-year-olds. Similarly, Feingold (1988) also found that the average DAT scores of boys increased steadily relative to those of girls in each year from 14 to 18 years of age for many intellectual abilities: verbal reasoning, abstract reasoning, numeric ability, space relations, and mechanical reasoning.

While a changing pattern from female superiority to male superiority appears to be the general finding in studies involving child and adolescent samples, a pattern of male superiority seems to be the dominant finding in studies involving adult samples. For example, Lynn (1994, 1999) found a male superiority of 4.0 IQ points in general intelligence in the adult data collected from a wide range of nations (e.g., Belgium, Britain, China, England, Estonia, Germany, Greece, Hawaii, Indonesia, Ireland, Israel, Japan, Norway, Portugal, Scotland, South Africa, Sweden, United States). Allik et al. (1999) even reported a male superiority of 11.4 IQ points in reasoning ability in an adult sample. Moreover, Colom et al. (2002) found a male superiority of 1.0 IQ point in the average IQ scores in a sample involving emerging adults.

### Extending the Study of the Developmental Theory of Sex Differences in Intelligence to the Creativity Domain

Extending the study of the developmental theory of sex differences in intelligence to the creativity domain may contribute to understanding the sex-creativity relationship, which has remained an unresolved research question in the field (Abraham, 2016). Some research findings suggest trivial sex differences in creativity (e.g., Kaufman et al., 2004; Matud et al., 2007), whereas others suggest significant sex differences in creativity, with either a female superiority (e.g., Wolfradt and Pretz, 2001; Reuter et al., 2005) or a male superiority (e.g., Dollinger et al., 2005; He et al., 2013). The developmental theory of sex differences in intelligence is insightful for illuminating the sex-creativity relationship because it offers a theoretical perspective for understanding the dynamic nature of sex differences in intellectual abilities. This theory suggests that sex differences in intellectual abilities may change in magnitude and even in direction with age. Such a theoretical perspective postulates that the seemingly contradictory findings available in the literature regarding the sex-creativity relationship may be related to samples with a limited age range or with collapsed age groups. For example, a study with a sample younger than 16 years of age may demonstrate trivial sex differences or small sex differences (with a female superiority), whereas a study with a sample older than 16 years of age may reveal a male superiority (with the effect size increasing across age). However, a study that consists of a sample with collapsed age groups may find no clear patterns of sex differences. In fact, researchers have increasingly recognized the merits of adopting a developmental approach to examining sex differences in cognitive functioning (e.g., Hyde, 2005; Arden and Plomin, 2006; Eriksson et al., 2012; He et al., 2015).

In the existing literature, few studies have examined the sex-creativity relationship from a developmental perspective. Recently, He et al. (2015) investigated the sex-creativity relationship in a sample that involved four age groups (i.e., 3–7, 9–13, 14–18, and 19–23 years). Their findings lent preliminary (though partial) empirical support to the developmental theory of sex differences in intelligence in the creativity domain. Consistent with the theory, they found a female superiority in the 3–7 age group, in which girls were overrepresented in the upper regions of the creativity score distribution; moreover, they found a male superiority in the 19–23 age group, in which males were overrepresented in the upper regions of the creativity score distribution. However, in the 9–13 and 14–18 age groups, they found that males were overrepresented in both the upper and lower regions (and females were overrepresented in the central region), suggesting that neither a female superiority nor a male superiority was found in the adolescent groups.

In addition to the significant sex differences that He et al. (2015) could find in various regions of the creativity score distribution (i.e., the upper and lower tails and the central region), it is interesting to note that they could find only trivial sex differences in the mean creativity scores in nearly all of the age groups (except the child group, in which girls significantly outperformed boys). In other words, He et al. (2015) obtained paradoxical findings with respect to the sex-creativity relationship based on mean analysis (i.e., sex differences in the mean creativity scores) and variability analysis (i.e., sex differences in various regions of the creativity score distribution). Such findings may show only partial support to the developmental theory of sex differences in intelligence in the creativity domain.

### The Present Study

#### Extending the Study of He et al. (2015) by Using a Longitudinal Design

While He et al. (2015) appeared to lend some empirical support to the developmental theory of sex differences in intelligence in the creativity domain, their study has a key limitation due to its cross-sectional design, in which they assessed sex differences in creative thinking in four different cohorts (i.e., 3–7, 9–13, 14–18, and 19–23 years). Researchers have fully recognized the methodological limitation of a cross-sectional design, which may hinder the reliability of revealing a genuine developmental pattern (Kleibeuker et al., 2013). The findings generated from a cross-sectional study may result only from a cohort effect (He and Wong, 2015), and a cohort effect can be related to many confounding variables (e.g., changes in environments or policies; Miller and Halpern, 2014).

The first aim of the present research was to extend He et al. (2015) by using a longitudinal design to study the developmental pattern of sex differences in creativity. As discussed in Section “The Developmental Theory of Sex Differences in Intelligence,” the developmental theory of sex differences in intelligence postulates a general trend with respect to the developmental pattern of sex differences, which suggests a changing pattern from small sex differences (favoring females) in childhood and early adolescence (during the age range of 8–15 years) to increasing sex differences (favoring males) from the age of 16 years onwards (Lynn, 1999; Lynn et al., 2000). This overall developmental trend suggests three different specific developmental patterns of sex differences in relation to three different developmental stages (i.e., childhood, adolescence, and adulthood). In childhood (when children grow from 8 to 11 years), a developmental trend of trivial sex differences (favoring females) is expected across ages. In adolescence (when adolescents grow from 14 to 17 years), a developmental trend with a change in direction from a small female superiority to a small male superiority starting at 16 years of age is expected. In emerging adulthood (when emerging adults grow from 18 to 22 years), a developmental trend of increasing male superiority is expected across ages.

**FIGURE 1:**
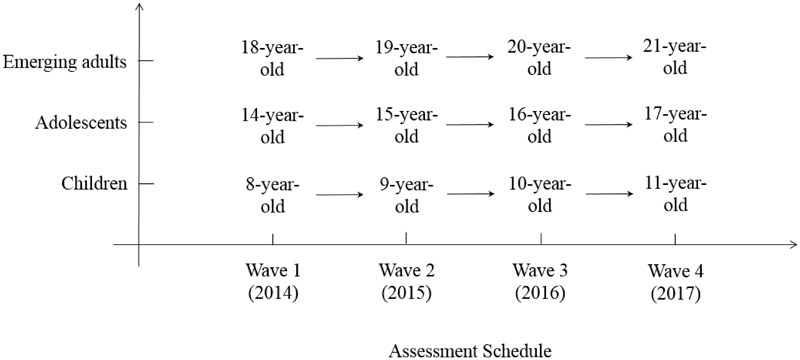
A diagrammatic representation of the 4-year longitudinal design, which consists of four waves of assessment in children, adolescents, and emerging adults.

It is our intention to investigate if these three postulated developmental patterns of sex differences in creative thinking are observed in their corresponding developmental stages. To achieve this study aim, we conducted a 4-year longitudinal study of sex differences in three age groups, namely a child group (growing from 8 to 11 years), an adolescent group (growing from 14 to 17 years), and an emerging adult group (growing 18 to 21 years). See Figure 1 for a diagrammatic representation of the study design, which shows that four waves of data were collected in four consecutive years from 2014 to 2017, with a one-year interval in between. By collecting the four years of longitudinal data in each of these age groups, we aimed to reveal the specific developmental patterns of sex differences in creative thinking that is associated with each developmental stage (i.e., childhood, adolescence, and emerging adulthood). See Table 1 for a summary of the predicted patterns of sex differences at the four waves of data collection across the 4-year longitudinal study in the three age groups.

**Table 1:** The age, education, and predicted patterns of sex differences at the four waves of data collection from 2014 to 2017 in the three age groups.

	Age			Predicted sex differences	
Assessment schedule	*Mean*	*SD*	Education	Small sex differences (favoring females)	Sex differences (favoring males)
**Children (*n* = 272)**					
Wave 1 (2014)	8.30	0.45	Grade 3	✓	
Wave 2 (2015)	9.20	0.48	Grade 4	✓	
Wave 3 (2016)	10.3	0.47	Grade 5	✓	
Wave 4 (2017)	11.5	0.49	Grade 6	✓	
**Adolescents (*n* = 265)**					
Wave 1 (2014)	14.4	0.41	Grade 9	✓	
Wave 2 (2015)	15.3	0.41	Grade 10	✓	
Wave 3 (2016)	16.3	0.42	Grade 11		✓
Wave 4 (2017)	17.3	0.42	Grade 12		✓
**Emerging adults (*n* = 238)**					
Wave 1 (2014)	18.4	0.70	Year 1		✓
Wave 2 (2015)	19.4	0.69	Year 2		✓
Wave 3 (2016)	20.7	0.71	Year 3		✓
Wave 4 (2017)	22.1	0.71	Year 4		✓

#### Analyzing the Sex-Creativity Relationship Based on Both Mean and Variability Analyses

Whereas the findings by He et al. (2015) based on variability analysis provided empirical support to the developmental theory of sex differences in intelligence in both the child and emerging adult groups, their findings based on mean analysis offered empirical support to the theory in only the child group. In fact, these seemingly paradoxical findings are not unusual because mean analysis and variability analysis concern different aspects of sex differences (Hyde and Mertz, 2009). While mean analysis reveals the sex differences in the central tendencies of test scores, variability analysis reveals the sex differences in the distributions of test scores. Specifically, mean analysis concerns the sex differences in the mean scores, whereas variability analysis concerns the sex differences in the variance ratios of the two sexes or the sex ratios (i.e., male/female ratios) in various regions of the score distributions, including the upper and lower tails (i.e., the higher and lower score regions) and the central region (i.e., the moderate score region).

Increasingly more research has shown that the findings derived from mean or variability analyses could reveal distinct or contradictory patterns of sex differences (e.g., He and Wong, 2011, 2014; He et al., 2013, 2015; Karwowski et al., 2016b). Hence, aiming to enhance understanding about the sex-creativity relationship, researchers suggest that it is essential to analyze sex differences based on both mean and variability analyses (He and Wong, 2011; He et al., 2013, 2015). In this connection, the second aim of this study was to employ both mean and variability analyses to reveal the developmental pattern of sex differences in creativity in three age groups.

#### The Hypotheses

Drawing on the developmental theory of sex differences in intelligence, we formulated three hypotheses with respect to the developmental patterns of sex differences in creativity during three different developmental stages (see Table 1 for a summary of the hypotheses).

Hypothesis 1. The child group (growing from 8 to 11 years over the 4-year span of the study) will demonstrate small sex differences (favoring females) across the four waves of the assessments from 2014 to 2017.

Hypothesis 2. The adolescent group (growing from 14 to 17 years over the 4-year span of the study) will demonstrate a changing pattern from trivial or small sex differences (favoring females) at 14–15 years of age (Wave 1–2) to a male superiority at 16–17 years of age (Wave 3–4).

Hypothesis 3. The emerging adult group (growing from 18 to 21 years over the 4-year span of the study) will demonstrate an increasing male superiority across the four waves of the assessments from 2014 to 2017.

## Materials and Methods

### Participants and Procedure

A total of 985 individuals participated at baseline. Ultimately, 78.7% of them (i.e., 775 participants) completed all four waves of the assessment, and the overall attrition rate was 21.3%. The final sample consists of three age groups: 272 children (49.6% female; attrition rate = 16.8%), 265 adolescents (50.6% female; attrition rate = 19.7%), and 238 emerging adults (50.8% female; attrition rate = 27.4%). Table 1 presents the mean ages and educational levels of these three groups across the four waves of the data collection. The child group and the adolescent group were recruited from three primary and secondary schools in Hong Kong, respectively. With respect to the emerging adult group, they were recruited from two universities in Hong Kong. All of the participating schools receive full subvention from the Hong Kong government.

According to the information provided by the participating schools in the children and adolescents groups, and the self-reported information provided by the emerging adults, all of the participating students belonged to the Chinese ethnic group, and they were mostly from middle-class to lower-middle-class socio-economic backgrounds. Socio-economic backgrounds were indicated by parents’ level of education. See Table 2 for the summary statistics regarding the highest level of education attained by the parents in the three age groups. Generally, most parents (53–62%) had obtained an education level of secondary school, and a few of them (13–24%) had obtained a tertiary level of education. No statistically significant differences were found between the two sexes in terms of their parents’ education level (all χ^2^ values ≤ 0.84, *p*-values ≥ 0.82).

**Table 2:** Parental education level of the male and female participants in the three age groups at baseline, and the results of the χ^2^ test (df = 3).

	Children		Adolescents		Emerging adults	
	*Male* (*n* = 137)	*Female* (*n* = 135)	*Male* (*n* = 131)	*Female* (*n* = 134)	*Male* (*n* = 117)	*Female* (*n* = 121)
**Father’s education level**						
Postgraduate	4%	3%	2%	3%	3%	4%
University	24%	22%	19%	20%	16%	15%
Secondary	54%	59%	58%	60%	58%	62%
Primary	18%	16%	21%	17%	23%	19%
*Results of the χ^2^ test* (*df = 3*)	(χ^2^ = 0.59, *p* = 0.90)		(χ^2^ = 0.78, *p* = 0.82)		(χ^2^ = 0.84, *p* = 0.83)	
**Mother’s education level**						
Postgraduate	2%	3%	0%	1%	1%	1%
University	22%	23%	17%	18%	13%	15%
Secondary	56%	53%	57%	53%	58%	57%
Primary	20%	21%	26%	28%	28%	27%
*Results of the χ^2^ test* (*df = 3*)	(χ^2^ = 0.33, *p* = 0.95)		(χ^2^ = 0.88, *p* = 0.84)		(χ^2^ = 0.25, *p* = 0.97)	

An exploration of the thinking process was explained as the main objective of the present study. Written informed consent was obtained from all participants. Furthermore, written informed consent was also obtained from the parents of the participants who were under the age of 18. All participants joined on a voluntary basis. The participants were assured that all information gathered during the study would be kept strictly confidential and that it would be used only for research purposes. The creativity test (i.e., TCT-DP) was administered in a group setting with standard instructions; approximately 20–35 participants were tested at a time.

### Instrument

Following He et al. (2015), we employed the Test for Creative Thinking-Drawing Production (TCT-DP, Urban and Jellen, 1995/2010) to assess creative thinking in the present study. The TCT-DP was developed based on a holistic and gestalt-oriented conceptualization of creativity (Urban and Jellen, 1995/2010; Urban, 2004). The test assesses creativity with a drawing task on an A4-sized testing sheet containing six intriguing figural fragments: (a) a semicircle, (b) a point (c) a 90° angle, (d) a curved line, (e) a broken line, and (f) a small open square. The drawing can be completed using any combination of the six figural fragments in a wide variety of ways ranging from simple, conventional, and disjointed completions to thematically complex, unconventional, integrated, and esthetically interesting completions (Dollinger et al., 2004).

The original TCT-DP contains two parallel forms: Forms A and B. Both forms contain the same elements, while Form B is the inversion (i.e., 180° rotation) of Form A. For the present study, creativity was measured at four time points, and four parallel forms were needed. Therefore, two additional forms, referred to as Form C (a version of Form A rotated 90°) and Form D (a version of Form A rotated 270°), were prepared according to the same principles as Forms A and B. The standard instructions for administering the TCT-DP were adapted and translated into Chinese via a back-translation procedure. Based on the TCT-DP test manual, creative thinking was scored according to 10 criteria (see Table 3 for a summary of the scoring criteria). A composite score was obtained by summing the points that were scored for each of the 10 criteria with no transformation. The total possible score range of the TCT-DP is 0–66 points, with a higher score indicating a better performance on the test.

**Table 3:** Scoring criteria of the TCT-DP.

Criterion	Descriptions	Score range
(1) Continuations	Any use or extension of the six fragments	0–6
(2) Completions	Any additions to the six continuations	0–6
(3) New elements	Any new figures or symbols added to the drawing	0–6
(4) Connections that are made with a line (Connections[Line])	Any physical linkages between the continuations or completions of the given fragments and the New elements	0–6
(5) Connections made to produce a theme (Connections[Theme])	Any elements or figures that contribute to a compositional theme	0–6
(6) Boundary breaking [Fragment-dependent]	Any uses of the small open square that is located outside of the large square frame	0–6
(7) Boundary breaking [Fragment-independent]	Any non-accidental drawing outside of the frame, excluding the use of the small open square	0–6
(8) Perspective	Any inclusions of the three-dimensional compositional whole or elements	0–6
(9) Humor and affectivity	Any expressions of humor or other emotions	0–6
(10) Unconventionality	Consists of the four subcategories below: (a) manipulations of the materials (b) surreal or abstract drawings (c) atypical combinations of figures and symbols (d) non-stereotypical use of a certain element	0–3 0–3 0–3 0–3

*The final criterion, *Speed*, was not applied in this study because the test was administered in group mode.*

The TCT-DP has increasingly been recognized as a promising instrument to assess creative thinking in a great variety of age groups from children to elders (Davis, 1995; Cropley, 2000; Wong et al., 2014). Its psychometric properties such as internal consistency, inter-rater reliability, criterion validity, convergent validity, and discriminant validity have been reported in a number of studies (see Urban and Jellen, 1995/2010; Dollinger et al., 2004; Rudowicz, 2004; Urban, 2004; Lubart et al., 2010). For example, several studies have supported its validity by showing significant correlations between the TCT-DP and a wide range of well-established creativity measures pertaining to different aspects of creativity, including divergent thinking (e.g., Torrance Test of Creative Thinking; Torrance, 1998), creative achievement (e.g., Creativity Behavior Inventory; Hocevar, 1979), and creative personality (e.g., Openness Scale of the NEO-Five Factor Inventory; Costa and McCrae, 1992). The applicability of the instrument in Chinese samples has also been supported in many studies (Rudowicz, 2004; He and Wong, 2011, 2015; He et al., 2013, 2017a,b). In this study, reasonably good internal consistency statistics were obtained, and the Cronbach’s alphas of Forms A, B, C, and D were 0.78, 0.79, 0.77, and 0.75, respectively. Moreover, an inter-rater reliability analysis using Pearson’s correlation was performed by having two experienced raters blind to the study score 240 TCT-DP protocols (i.e., 31.0% of the sample), with 60 protocols from each of the four test forms. A high inter-rater correlation coefficient was obtained for the composite score of the TCT-DP (*r* = 0.94; *p* < 0.001), which is comparable to the values reported in the test manual (*r* = 0.89–0.97, Urban and Jellen, 1995/2010).

### Data Analysis

Sex differences were analyzed based on both mean and variability analyses. In mean analysis, a male or female superiority was indicated by a higher mean TCT-DP score. To examine mean differences, a 2 (sex groups) × 4 (time points) repeated-measures analysis of covariance (ANCOVA) was applied to determine if the following effects were statistically significant after the possible covariate effect of socio-economic background was controlled: (1) the main effect of sex (i.e., whether there were statistically significant sex differences in the mean TCT-DP scores); (2) the main effect of time (i.e., whether there were statistically significant changes in the TCT-DP scores across the four time points); and (3) the interaction effect of Sex × Time (i.e., whether there statistically significant sex differences in the longitudinal changes of the TCT-DP scores across the four time points).

In variability analysis, a male or female superiority was measured with the male/female ratios in various regions of the TCT-DP score distribution (i.e., the upper and lower tails and the central region). A male/female ratio greater than 1.0 indicated that more males fell into a certain region, whereas a male/female ratio smaller than 1.0 indicated that more females fell into a certain region. Reasonably, a male superiority was indexed with more (and fewer) males falling into the higher (and lower) score regions. Similarly, a female superiority was indexed with more (and fewer) females falling into the higher (and lower) score regions. Chi-square tests were applied to test for significant differences in the sex composition in various regions of the TCT-DP score distribution. Because of the slight but nonsignificant difference in the gender proportions of the sample in each of the three age groups (children: 49.6% females, χ^2^[1, *N* = 272] = 0.02, *n.s.*; adolescents: 50.6% females, χ^2^[1, *N* = 265] = 0.03, *n.s.*; emerging adults: 50.8% females, χ^2^[1, *N* = 238] = 0.07, *n.s.*), the male/female ratios in various regions of the score distribution were calculated using the percentage scores within each gender to adjust for the minor gender imbalance of the sample.

## Results

**FIGURE 2:**
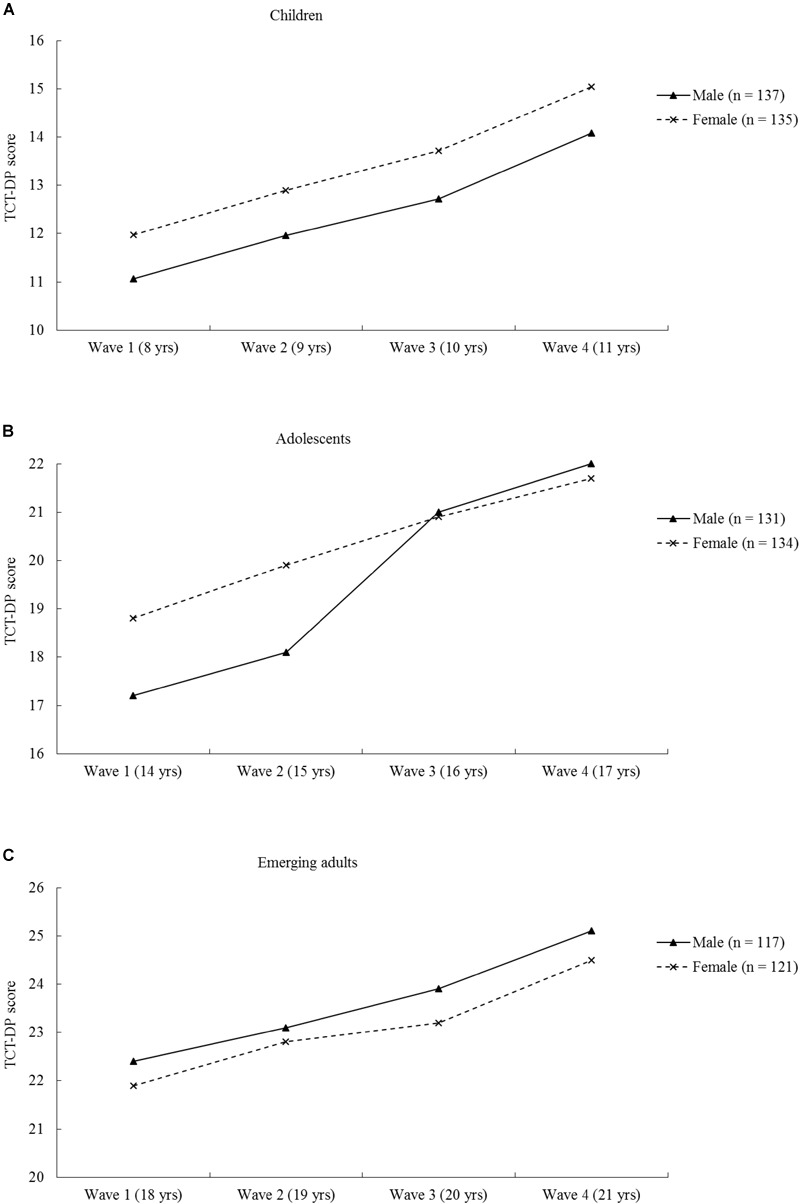
The 4-year longitudinal trend of sex differences in creative thinking in the age groups of **(A)** children (growing from 8 to 11 years old), **(B)** adolescents (growing from 14 to 17 years old), and **(C)** emerging adults (growing from 18 to 21 years old).

Prior to testing the hypotheses, it is necessary to test the normality assumption of the data obtained in this study. The results based on the Shapiro–Wilk test (Razali and Wah, 2011) suggest that the data of the four waves of assessment in the three age groups were normally distributed in both sexes, with the *W*-values ranging between 0.967 and 0.986 (all *p-*values > 0.05). Hence, the normality assumption is met and relevant parametric tests can be used for testing the hypotheses. In the subsequent sections, the results of the hypotheses testing based on both mean and variability analyses are summarized according to the three hypotheses with reference to the three different developmental patterns of sex differences in creativity in each of the age groups. Results in relation to the mean analysis are shown in Figure 2 and Tables 4, 5, while statistics with respect to the variability analysis are described in Table 6.

**Table 4:** Means and standard deviations (SD) of the TCT - DP scores obtained by males and females.

	Males		Females	
	*Mean*	*SD*	*Mean*	*SD*
**Children**	*n* = 137		*n* = 135	
Wave 1 (8 years)	11.1	3.56	12.0	3.78
Wave 2 (9 years)	12.0	3.39	12.9	4.19
Wave 3 (10 years)	12.7	3.90	13.7	4.30
Wave 4 (11 years)	14.1	4.27	15.0	3.57
**Adolescents**	*n* = 131		*n* = 134	
Wave 1 (14 years)	17.2	7.95	18.8	5.27
Wave 2 (15 years)	18.1	8.30	19.9	6.28
Wave 3 (16 years)	21.0	9.05	20.9	5.24
Wave 4 (17 years)	22.0	9.86	21.7	7.01
**Emerging adults**	*n* = 117		*n* = 121	
Wave 1 (18 years)	22.4	8.98	21.9	5.69
Wave 2 (19 years)	23.1	10.3	22.8	6.02
Wave 3 (20 years)	23.9	10.4	23.2	6.59
Wave 4 (21 years)	25.1	11.7	24.5	6.49

**Table 5:** Results of repeated measures ANCOVAs.

Source	*df*	*F*-value	$\eta_{p}^{2}$ value	Wilks’ lambda
**Children**				
Sex	*df* (1, 270)	5.43*	0.02	–
Time	*df* (3, 268)	97.9***	0.52	0.48
Sex × Time	*df* (3, 268)	0.02	0.00	1.00
**Adolescents**				
Sex	*df* (1, 263)	0.93	0.00	–
Time	*df* (3, 261)	27.1***	0.24	0.77
Sex × Time	*df* (3, 261)	2.72*	0.03	0.97
**Emerging adults**				
Sex	*df* (1, 236)	0.26	0.00	–
Time	*df* (3, 234)	28.8***	0.27	0.73
Sex × Time	*df* (3, 234)	0.09	0.00	1.00

*^∗^*p* < 0.05; ^∗∗∗^*p* < 0.001.*

**Table 6:** Male/female ratios (sex ratios) and results of the variability analysis.

	*z* ≤- 2.0		- 2.0 *< z* ≤-1.0		-1.0 < *z* < 1.0		*1 ≤ z < 2.*0		*z* ≥ 2.0	
	Sex ratio	*χ*^2^	Sex ratio	*χ*^2^	Sex ratio	*χ*^2^	Sex ratio	*χ*^2^	Sex ratio	*χ*^2^
**Children (*n* = 272)**					
Wave 1 (8 years)	7.29	4.41^∗^	2.96	9.68***	0.87	0.83	0.58	1.93	0.13	4.60*
Wave 2 (9 years)	9.42	6.27^∗^	2.94	6.78**	0.94	0.22	0.46	2.69	0.10	6.53*
Wave 3 (10 years)	8.29	5.33^∗^	3.06	11.4***	0.89	0.63	0.51	1.93	0.13	4.60*
Wave 4 (11 years)	9.43	6.27^∗^	2.43	6.48**	0.88	0.81	0.60	1.27	0.13	4.60*
**Adolescents (*n* = 265)**					
Wave 1 (14 years)	7.57	4.65^∗^	3.96	12.3***	0.74	4.32*	1.02	0.00	2.07	0.72
Wave 2 (15 years)	8.71	5.62^∗^	5.87	13.8***	0.74	4.60*	1.13	0.08	2.53	1.36
Wave 3 (16 years)	7.57	4.65^∗^	3.05	8.39**	0.65	8.40**	2.50	4.41*	3.45	3.94*
Wave 4 (17 years)	8.71	5.62^∗^	3.22	7.08*	0.62	9.93**	2.21	5.56*	4.07	3.95*
**Emerging adults (*n* = 238)**					
Wave 1 (18 years)	7.50	4.70^∗^	5.80	10.3***	0.64	8.61**	3.34	6.12*	4.53	4.68*
Wave 2 (19 years)	7.50	4.70^∗^	3.24	7.18**	0.63	8.96**	3.36	5.06*	5.00	5.59*
Wave 3 (20 years)	7.50	4.70^∗^	3.24	7.18**	0.61	10.0***	4.04	7.72*	5.53	6.52*
Wave 4 (21 years)	7.50	4.70^∗^	7.53	10.4***	0.61	10.3***	5.60	9.88**	6.53	8.43**

*A sex ratio greater than 1.0 denotes more males fall into a certain region, whereas a sex ratio smaller than 1.0 denotes more females fall into a certain region. ^∗^*p* < 0.05; ^∗∗^*p* < 0.01; ^∗∗∗^*p* < 0.001.*

### Hypothesis 1 - Small Sex Differences (With a Female Superiority) in the Child Group

#### Results of the Mean Analysis in the Child Group

As shown in Figure 2A and Table 4, female children appeared to obtain higher TCT-DP scores than their male counterparts at all four waves of assessments when they grew from 8 to 11 years over the 4-year span of the study. Table 5 presents the results of the repeated measures of ANCOVAs, which revealed that the Sex effect was significant, suggesting a statistically significant female superiority in this group. Furthermore, the results revealed a significant Time effect but a nonsignificant Time × Sex interaction effect, suggesting that both sexes showed improving performance in the creativity test across age in a parallel manner. The results regarding a significant female superiority over the 4-year span align with the prediction of Hypothesis 1.

#### Results of the Variability Analysis in the Child Group

As presented in Table 6, male/female ratios smaller than 1.0 were found for the higher score regions where z ≥ 2 (i.e., two standard deviations or more above the mean; male/female ratios = 0.10–0.13) and 1 ≤ z < 2 (i.e., one standard deviation or more above the mean; male/female ratios = 0.51–0.60) as well as the moderate score region (i.e., -1.0 < z < 1.0; male/female ratios = 0.87–0.94), suggesting an overrepresentation of females in the higher and moderate score regions. In the lower score regions, however, the male/female ratios for the regions where - 2 *< z* ≤- 1 (i.e., one standard deviation or more below the mean; male/female ratios = 2.43–3.06) and z ≤ –2 (i.e., two standard deviations or more below the mean; male/female ratios = 7.29–9.43) were all greater than 1.0, suggesting an overrepresentation of males in these lower score regions. The results of the chi-square tests further suggest that female participants significantly outnumbered male participants in the higher score region where z ≥ 2 while male participants significantly outnumbered female participants in the lower score regions where z ≤ –2 and - 2 *< z* ≤- 1, with all χ^2^ values ≥ 4.41, *p*s < 0.05. These results also tend to align with Hypothesis 1.

### Hypothesis 2 - A Changing Pattern From Small Sex Differences (With a Female Superiority) to a Male Superiority in the Adolescent Group

#### Results of the Mean Analysis in the Adolescent Group

As shown in Figure 2B and Table 4, male and female adolescents did not show a consistent pattern of sex differences across the four waves of assessments during the 4-year span. The results summarized in Table 5 showed the overall Sex effect was not significant in this age group. However, the Time effect and the Time × Sex effect were both significant. The significant Time effect suggests that both male and female adolescents improved in their creative thinking across age, while the significant Time × Sex effect implies that the two sex groups actually exhibited different developmental trends in their mean TCT-DP scores across age during the adolescence years.

Hence, subsequent repeated-measures ANCOVAs were further performed separately for the two sex groups to analyze their specific developmental patterns. An adjusted *p* value of 0.025 (i.e., 0.05/2) was used to determine the significance level of the statistical tests. Results revealed that female adolescents showed a steady and statistically significant improvement in their mean TCT-DP scores through Wave 1 to Wave 2 (from 14 to 15 years old; *F*[1,133] = 8.41, *p* < 0.01, $\eta_{p}^{2}$ value = 0.06) and Wave 3 (16 years old; *F*[1,133] = 8.41, *p* < 0.01, $\eta_{p}^{2}$ value = 0.06). However, they showed no further significant improvement in their mean TCT-DP scores from Wave 3 to 4 (17 years old; *F*[1,133] = 2.45, *p* = 0.12, $\eta_{p}^{2}$ value = 0.02). Showing a different developmental trend, male adolescents exhibited a slow and non-significant improvement in their mean TCT-DP scores between Waves 1 and 2 (from 14 to 15 years old; *F*[1,130] = 3.11, *p* = 0.08, $\eta_{p}^{2}$ value = 0.02), followed by a rapid and significant improvement through Wave 2 (15 years old) to Wave 3 (16 years old; *F*[1,130] = 19.91, *p* < 0.001, $\eta_{p}^{2}$ value = 0.13) and Wave 4 (17 years old; *F*[1,130] = 7.26, *p* < 0.01, $\eta_{p}^{2}$ value = 0.03).

The distinct developmental patterns between male and female adolescents suggest that different patterns of sex differences may appear at different time points across the four waves of assessments. This speculation was supported by the results of a subsequent univariate ANCOVA, which illustrated that the significant sex differences (favoring females) were only observed at Wave 1 (14 years old; *F*s[1,263] = 4.04, *p* < 0.05, $\eta_{p}^{2}$ value = 0.02) and Wave 2 (15 years old; *F*s[1,263] = 3.88, *p* < 0.05, $\eta_{p}^{2}$ value = 0.02); however, no significant sex differences were found at Wave 3 (16 years old; *F*s[1,263] = 0.00, *p* = 0.96, $\eta_{p}^{2}$ value = 0.00) and Wave 4 (17 years old; *F*s[1,263] = 0.11, *p* = 0.75, $\eta_{p}^{2}$ value = 0.00). These results with respect to a changing pattern from a female superiority (at 14–15 years) to negligible sex differences (at 16–17 years) were not in line with Hypothesis 2.

#### Results of the Variability Analysis in the Adolescent Group

Referring to Table 6, an interesting changing pattern was also observed in the higher score regions (i.e., z ≥ 2 and 1 ≤ z < 2) for this age group. Specifically, in the higher score region where z ≥ 2, the male/female ratios increased steadily across the four waves from 2.07 (at Wave 1) to 4.07 (Wave 4); a male/female ratio greater than 1.0 suggests an overrepresentation of males in this region. Furthermore, in another higher score region where 1 ≤ z < 2, a somewhat similar increasing trend of overrepresentation of males was also observed, with the male/female ratios increasing from 1.02 (at Wave 1) to 2.21 (at Wave 4). This increasing trend suggests that increasingly more males (but increasingly fewer females) fell into the higher score regions across age during adolescence. While the results of the chi-square tests suggest that the overrepresentation of males over females in the higher score regions did not reach a significant level at Waves 1 and 2 (*Mean* age = 14.4–15.3 years; χ^2^ values ranged between 0.00 and 1.36, *n.s.*), the overrepresentation of males over females reached a significant level at Waves 3 and 4 (*Mean* age = 16.3–17.3 years old; χ^2^ values ranged between 2.21 and 4.07, *p*s < 0.05). These results suggest a changing pattern from a non-significant overrepresentation of males to a statistically significant overrepresentation of males in the high tail beginning at approximately 16 years of age.

In contrast to the trend of an increasing male/female ratio in the higher score regions, an interesting opposite trend (i.e., a decreasing male/female ratio) was observed in the moderate score region (i.e., –1.0 < z < 1.0), in which the male/female ratio decreased from 0.74 (at Waves 1 and 2) to 0.65 (at Wave 3) and 0.62 (at Wave 4). While a male/female ratio smaller than 1.0 suggest an overrepresentation of females in this central region, a decreasing magnitude in the male/female ratio further suggests a changing trend in which increasingly more females (but increasingly fewer males) fell into the moderate score region across age in adolescence. The results of chi-square tests further supported a significant overrepresentation of females in the moderate score region across the four waves of assessment (all χ^2^ values ≥ 4.32, *p*s < 0.05).

In contrast to the changing patterns in the upper and central regions, no systematic changing pattern in the lower score regions was found (i.e., z ≤ –2 and - 2 *< z* ≤- 1); however, the results of the chi-square tests revealed a somewhat consistent and significant overrepresentation of males in these lower score regions, with all male/female ratios greater than 1.0 (for z ≤ –2: male/female ratios = 7.57–8.71; for - 2 *< z* ≤- 1: male/female ratios = 3.05–5.87; all χ^2^ values ≥ 4.32, *p*s < 0.05). Overall, the results of the variability analysis in the adolescent group generally suggest that across age, an increasing trend of overrepresentation of males and females was observed in the higher and the moderate score regions, respectively; however, in the lower score regions, a somewhat consistent pattern of overrepresentation of males was observed. Similar to the findings based on the mean analysis, these results generated from the variability analysis were not entirely in line with Hypothesis 2.

### Hypothesis 3 - An Increasing Male Superiority in the Emerging Adult Group

#### Results of the Mean Analysis in the Emerging Adult Group

The statistics shown in Figure 2C and Table 4 seem to suggest a consistent male superiority in this age group over the 4-year span of the study. However, the results of the repeated-measures ANCOVA suggest that the Sex effect was actually not significant (Table 5), implying that the sex differences in this age group were too trivial to reach a statistically significant level. The results of the repeated-measures ANCOVA further revealed a non-significant Time × Sex effect but a significant Time effect, which suggests that both sexes showed a steady and significant improvement in their mean TCT-DP scores in a parallel manner across age and no significant sex differences were found at any time point of the four waves of assessments. In other words, the results obtained in this age group were not consistent with Hypothesis 3, which posits an increasing male superiority across age in adulthood.

#### Results of the Variability Analysis in the Emerging Adult Group

Regarding variability analysis, it was interesting to find a significant overrepresentation of males in the higher score regions (i.e., 1 ≤ z < 2 and z ≥ 2; all χ^2^ values ≥ 4.68, *p*s < 0.05) across Waves 1 and 4. However, in the moderate score region (i.e., –1.0 < z < 1.0), a reverse pattern, i.e., a significant overrepresentation of females, was consistently found at all four waves of assessment (all χ^2^ values ≥ 8.61, *p*s < 0.01). Even more interestingly, it was found that the male/female ratios increased steadily from Wave 1 to Wave 4 in the higher score regions (for z ≥ 2: male/female ratios increase from 4.53 to 6.53; for 1 ≤ z < 2 : male/female ratios increase from 3.34 to 5.60). However, in the moderate score region (i.e., –1.0 < z < 1.0), the male/female ratios decreased gradually from 0.64 (at Wave 1) to 0.61 (at Wave 4).

In the lower score regions, the results of the chi-square tests revealed a somewhat consistent and significant overrepresentation of males (for z ≤ –2: male/female ratios = 7.50; for - 2 *< z* ≤- 1: male/female ratios = 3.24–7.53; all χ^2^ values ≥ 4.70, *p*s < 0.05). Overall, the results of the variability analysis in the emerging adults group appeared to suggest that while males and females were increasingly overrepresented in the higher and moderate score regions, respectively, males were consistently overrepresented in the lower score regions. Such results were not entirely consistent with Hypothesis 3, which postulated an increasing male superiority across age in the emerging adult group.

## Discussion

Understanding the developmental patterns of sex differences in intellectual abilities is a challenging task; however, it is a question that warrants research attention because such a question has important social, educational, and political implications (Toivainen et al., 2017). Despite years of research, sex differences in creativity remain an intriguing issue. The present study extended this line of research by (1) taking a developmental perspective, (2) employing a 4-year longitudinal design in three age groups, and (3) analyzing sex differences based on both mean and variability analyses. Some interesting and important findings are highlighted in the sections below.

### Mean Analysis Suggests a Developmental Pattern From a Small Female Superiority to Greater Sex Similarities

Cohen (1988) rule of thumb was applied to determine the effect size or the practical significance of the mean differences, in which a $\eta_{p}^{2}$ value of 0.01 (or more), 0.06 (or more), and 0.14 (or more) indicated a small, moderate, and large effect, respectively. Based on Cohen (1988) suggestion, the results of the mean analysis in the present study appeared to illustrate a developmental pattern from a small female superiority to trivial sex differences across age. Specifically, in the child group, female participants consistently obtained significantly higher mean TCT-DP scores than their male counterparts (through small effect sizes with an $\eta_{p}^{2}$ value = 0.02) when they grew from 8 to 11 years old. This pattern of a small female superiority in mean TCT-DP scores was further observed in the adolescent group until 15 years old (again, via small effect sizes with an $\eta_{p}^{2}$ value = 0.02). However, when this adolescent group reached the ages of 16 and 17, the sex-related pattern of a small female superiority disappeared and negligible sex differences were observed in the mean TCT-DP scores (with an $\eta_{p}^{2}$ value = 0.00). Such a pattern of negligible sex differences was further consistently observed in the emerging adult group in the years when they were aging from 18 to 21 years old ($\eta_{p}^{2}$ value = 0.00).

Hence, integrating the findings of the three age groups together appears to suggest an age-related pattern of sex differences in the mean TCT-DP scores: on average, a female superiority (with small effect sizes) in the mean TCT-DP scores were found before the age of 16, while no significant sex differences were found in the mean TCT-DP scores when the participants reached the age of 16 and onwards, in which the sex differences were nearly negligible with the effect size ($\eta_{p}^{2}$ value) being close to zero. The findings of trivial sex differences in the mean TCT-DP scores starting at 16 years of age appeared to be inconsistent with the prediction of the developmental theory of sex differences in intelligence which postulates an increasing male superiority beginning from the age of 16 and onwards (Lynn, 1999; Lynn et al., 2000). Rather, such findings appeared to be in line with the arguments of many researchers who have dissented from the view of great psychological sex differences (e.g., Hollingworth, 1918; Hyde, 2014). Relying on accumulated research evidence mainly based on meta-analyses, researchers have even argued for greater sex similarities and have postulated the gender similarities hypothesis (Hyde, 2005) to argue that males and females are similar in most (though not all) psychological attributes, including cognitive performance, personality and social behaviors (Hyde, 2014). Moreover, there is also much empirical evidence supporting that men and women have equal or nearly equal mean abilities in the domains of intellectual and cognitive functioning (Hyde et al., 2008; Ceci et al., 2009; Toivainen et al., 2017). Adding to this body of literature, the findings of this study, based on the mean analysis of the 4 years of longitudinal data of the three age groups, revealed that in the domain of creativity, the two sexes demonstrated nearly equal mean abilities starting at 16 years.

### Variability Analysis Revealed a Developmental Pattern From a Female Superiority to Greater Male Variability

Integrating the results of the variability analysis of the three age groups together yielded an interesting developmental pattern of sex differences in various regions of the TCT-DP score distribution (i.e., the higher, moderate, and lower score regions). In the child group, an overrepresentation of females was consistently observed in both the higher and moderate score regions, whereas an overrepresentation of males was predominant in the lower score regions across the ages from 8 to 11 years old, tending to support a female superiority. However, among the adolescents who were growing from 14 to 17 years, a female superiority was no longer observed. Rather, the results illustrated an overrepresentation of males in both the higher and lower score regions and a reverse pattern (i.e., an overrepresentation of females) in the moderate score region. This finding with respect to an excessive number of males occupying both the higher and lower extremes of the score levels, together with an excessive number of females occupying the moderate score level, appears to suggest that males demonstrate greater variability than their female counterparts in their performance on the creativity test. This pattern of greater male variability was further observed in the emerging adult group when the subjects were growing from 18 to 21 years.

Hence, the results of the variability analyses, in general, suggest a changing pattern from female superiority to greater male variability, which is not entirely consistent with the prediction of the developmental theory of sex differences in intelligence. Rather, the findings regarding a greater male variability tended to be in line with the greater male variability hypothesis (Ellis, 1894), which postulates that males are more variable in their abilities than females in many domains. Therefore, males usually demonstrate a wider distribution than females do. The greater male variability hypothesis accounts for the greater numbers of males falling at both the upper and lower extremes of the distribution of abilities (Feingold, 1992; Johnson et al., 2008). Such a hypothesis has been proven to be useful in understanding many real-world phenomena in which men are much more heavily represented than women at both the highest and lowest levels of ability or achievement in many domains or areas (Johnson et al., 2008; Hyde and Mertz, 2009; Hyde, 2014). Additionally, there has long been a real-world phenomenon in which “there are more male geniuses, more male criminals, (and) more male mental defectives in spite of only minor differences between the mean performances of the two sexes” (Lehre et al., 2008; p. 198). In scientific research, a large body of empirical findings has provided additional support to the greater male variability hypothesis in a wide range of psychological attributes (Hedges and Nowell, 1995; Machin and Pekkarinen, 2008; Lakin, 2013; He and Wong, 2014), including creative thinking (He and Wong, 2011; He et al., 2013, 2015). Adding to this body of literature, the results of the variability analyses on the 4 years of longitudinal data of the three age groups revealed that in the domain of creativity, greater male variability emerged starting at 14 years of age.

More interestingly, the results of the present study further revealed that the magnitude of the male/female ratios in the high score regions increased steadily across age whereas a general decreasing pattern was found in the moderate score region (see Table 6). For example, in the high score region (where z ≥ 2), the male/female ratios increased steadily from 0.13 (at the age of 8) to 2.07 (at the age of 14) and 3.45 (at the age of 16), ultimately reaching 6.53 (at the age of 21). However, in the moderate score region (i.e., - 1.0 < *z* < 1.0), the male/female ratios decreased steadily from 0.87–0.94 in childhood to 0.61–0.64 in emerging adulthood. The changing pattern in the magnitude of the male/female ratios across age in the high and moderate score regions, in general, suggests that the spread of males’ scores becomes more and more sporadic whereas females’ scores become tighter and tighter around the mean. Would these findings suggest the possibility that some males make vast improvements while females stay the same or well behind across age? This sounds an interesting research question for further investigations.

### While Female Superiority Was Found in Childhood and Early Adolescence, No Male Superiority Was Found in Adolescence and Emerging Adulthood

Overall, the mean and variability analyses seemed to generate both homogeneous and heterogeneous findings with respect to the testing of the developmental theory of sex differences in intelligence in the creativity domain. With respect to the homogeneous findings, both mean and variability analyses illustrated that (1) the sex differences in creative thinking fluctuated with age; (2) the adolescent years (i.e., 14–16 years) appeared to be the critical period of a dramatic change in sex differences (in both magnitude and direction); and (3) a female superiority was found in childhood and early adolescence. These findings are somewhat in agreement with the developmental theory of sex differences in intelligence which postulates a female superiority before the age of 15.

With respect to the heterogeneous findings, the mean and variability analyses illustrated different sex-related patterns starting in adolescence. On one hand, mean analysis did not show significant sex differences in the mean TCT-DP score (i.e., trivial sex differences or greater sex similarities) starting at 16 years of age. On the other hand, variability analysis showed significant sex differences in the TCT-DP score distribution (i.e., greater male variability), with an over representation of male scoring in both the upper and lower tails. Both findings with respect to greater sex similarities and greater male variability suggest that, in general, males never catch up with females, which are not consistent with the prediction of the developmental theory of sex differences in intelligence regarding a male superiority starting from 16 years of age.

In summary, the findings of this longitudinal investigation were not entirely consistent with the prediction of the developmental theory of sex differences in intelligence. While this theory received substantial empirical support in the intelligence domain (see Lynn and Kanazawa, 2011, for a review), the findings that this theory received only partial support in the creativity domain may imply that this theory may be domain-specific. The findings with respect to the different types of applicability of this theory in the intelligence and creativity domains seem accord with many recent empirical findings suggesting that creativity and intelligence were actually two different psychological constructs even though they showed some correlations with each other to some degree (e.g., Nusbaum and Silvia, 2011; Jauk et al., 2013; Karwowski et al., 2016a). These research findings may imply that an alternative developmental theory of sex differences in creativity is needed for a better understanding of the age-related patterns of the sex-creativity link.

### Limitations and Future Research

Some limitations of this study should be noted. One of these limitations concerns the research design. While the current study design allowed the collection of longitudinal data that revealed a 4-year developmental trend of sex differences in creative thinking in specific developmental stages (i.e., childhood, adolescence, and emerging adulthood), these data are insufficient to reveal a complete and compelling picture regarding the overall developmental changes in sex differences through childhood and adolescence to emerging adulthood. These data are not sufficient enough to inform our understanding with reference to the real factors that lead to developmental changes in sex differences in creativity because the findings are likely to be confounded by cohort differences. Further longitudinal investigations over a longer span of time should be conducted throughout the developmental stages from childhood and adolescence to adulthood. For example, by taking the reference from the longitudinal design of Lynn and Kanazawa (2011), a longitudinal investigation with four waves of assessment at ages 7, 11, 16, and 21 can be considered in future research. Although such a longitudinal study will take approximately 14 years to complete, it should have better control of the cohort effect and serve as a more compelling design to examine the developmental theory of sex differences in intelligence.

Besides the research design, we also note several other limitations of the study. First, although the possible confounding effect of socio-economic background was controlled in the present study, socio-economic background was actually narrowly defined by parents’ education level; further careful empirical scrutiny is required to control for more confounding variables (e.g., personal characteristics, environmental variables, and socio-economic background as measured by family income and parents’ occupation) as much as possible with an aim to study the age effect in relation to the developmental patterns of sex differences, in which repeated-measures ANCOVAs should be used to adjust the effect of the seemingly confounding variables. Second, all participants of the study were Hong Kong Chinese students; future research should examine whether or not the findings can be generalized to other populations. Third, creativity was assessed with only a single measure of creativity (i.e., the TCT-DP); future research should address the question of whether the findings of this study can be replicated if other creativity tests are used. Fourth, although the TCT-DP is a well-established creativity test and many research findings support the applicability of test Forms A and B (e.g., He and Wong, 2011, 2015; He et al., 2013, 2017a,b), Forms C and D of this test were newly developed specifically for the third and fourth waves of assessment. Although the reliability of Forms C and D were equivalent to that of Forms A and B, the reliability and validity issues associated with this new form must be further addressed. Fifth, as suggested by He and Wong (2015), future research may also counterbalance the use of the TCT-DP forms (A, B, C, and D) with the time of testing to exclude the possibility that one of the forms may be more or less conducive to the participants’ creative performance. Finally, there might be a practice effect of the participants’ repeated performance on the TCT-DP test (though in four different forms) across the four time points; a negative effect could also arise from reduced participant motivation from performing the task repeatedly.

## Conclusion

Despite the abovementioned limitations, the findings derived from this 4-year longitudinal study and based on both the mean and variabilities analyses enrich our understanding of the developmental patterns of the sex-creativity link. In general, the findings revealed various patterns of sex differences or similarities across age: (1) the findings based on the mean analysis revealed female superiority and greater sex similarities before and starting at 16 years of age, respectively; (2) the findings based on the variability analysis revealed female superiority and greater male variability before and starting at 14 years of age, respectively. These seemingly paradoxical findings imply that the developmental trajectories of sex differences in intellectual abilities exhibit complex etiology, which may be related to (1) biological factors (e.g., Eysenck, 1995; Giummo and Johnson, 2008), (2) socio-cultural factors (e.g., Feingold, 1994; He et al., 2013), and (3) the interplay of bio-socio-cultural factors (e.g., Vernon, 1989; Wood and Eagly, 2002). Looking into the issue with multiple theoretical perspectives is helpful to understand the continuously changeable patterns of sex differences in intellectual abilities. Examples of some important theoretical perspectives in relation to the issue include the cognitive social learning theory (Bussey and Bandura, 1999), sociocultural theory (Wood and Eagly, 2002), expectancy-value theory (Eccles, 1994), and evolutionary theories (Buss and Schmitt, 1993).

## Ethics Statement

This study was carried out in accordance with the recommendations of the Operational Guidelines and Procedures of the Human Research Ethics Committee of the Education University of Hong Kong with written informed consent from all subjects. All subjects gave written informed consent in accordance with the Declaration of Helsinki. The protocol was approved by the Human Research Ethics Committee of the Education University of Hong Kong.

## Author Contributions

W-JH contributed to the conception and design of the work as well as the acquisition, analysis, and interpretation of the data. She also drafted, revised, and approved the final version of the manuscript. She agrees to be accountable for the content of the work.

## Conflict of Interest Statement

The author declares that the research was conducted in the absence of any commercial or financial relationships that could be construed as a potential conflict of interest. The reviewer YS and handling Editor declared their shared affiliation at time of review.
